# Spray coagulation as an alternative to argon plasma coagulation for bleeding portal hypertensive gastropathy

**DOI:** 10.1016/j.igie.2023.01.014

**Published:** 2023-02-27

**Authors:** Sahaj Rathi, Amandeep Singh, Virendra Singh

**Affiliations:** Department of Hepatology, Post Graduate Institute of Medical Education and Research, Chandigarh, India

## Abstract

Electrosurgical thermoablative modalities are commonly used for endoscopic hemostasis in nonvariceal sources of GI bleeding. Argon plasma coagulation (APC) is a noncontact type of thermoablative technique often used for treatment of bleeding gastric antral vascular ectasias and portal hypertensive gastropathy (PHG). However, its use depends on the availability of a dedicated APC unit, proprietary disposable probes, and the argon gas itself. This became a limitation in case of a 49 year old man with ethanol related cirrhosis of liver, who presented with diffusely bleeding PHG. In this situation, we did not have argon at hand, and improvised with an alternative technique of spray coagulation to successfully achieve hemostasis. Spray coagulation can potentially be used as an alternative to APC in mucosal bleeding.

Argon plasma coagulation (APC) is noncontact, monopolar, electrosurgical thermoablative modality often used to achieve hemostasis in surgical and endoscopic procedures. It involves the use of a high-voltage electrode that ionizes argon to create a high-temperature plasma arc leading to thermal coagulation of tissue in its proximity.^1^ Argon is an excellent choice of gas to generate plasma because of its inert nature, low breakdown voltage, and low cost.^2^ However, the use of this modality is contingent on the availability of argon gas, APC generator, and proprietary probes. Supply chain and procurement issues during the coronavirus disease 2019 pandemic often created shortages of crucial equipment on various occasions. This is a case of bleeding portal hypertensive gastropathy (PHG) where we ran out of argon gas, and improvised.

## Case Report

A 49-year-old man with ethanol-related cirrhosis of the liver presented with melena for 2 days. He had a history of GI bleeding, for which he had 2 sessions of endoscopic band ligation. He also had prior episodes of encephalopathy, for which he was on medical management with lactulose and rifaximin.

**Figure 1:**
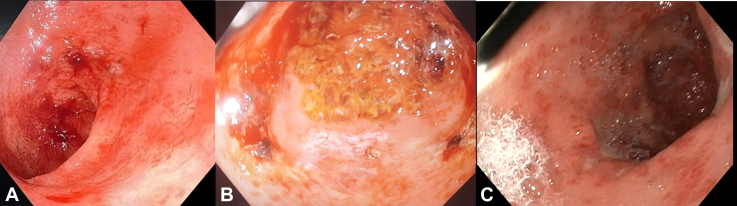
**A,** Initial endoscopic picture of bleeding portal hypertensive gastropathy. **B,** After the first session of spray coagulation. **C,** Relook endoscopy after 2 sessions of spray coagulation, 1 week from the last session.

At presentation he was hemodynamically stable. The laboratory workup showed a hemoglobin of 5.5 g/dL, white blood cell count of 6100/mm^3^, platelets of 102 × 10^3^/mm^3^, and an international normalized ratio of 1.58. He was volume resuscitated with crystalloids and a unit of packed red blood cells and was started on broad-spectrum antibiotics (ceftriaxone) and terlipressin. An EGD performed at this stage showed eradicated esophageal varices with severe PHG in the antropyloric region with ongoing hemorrhage (Fig. 1). Similar lesions were seen in the duodenal bulb, gastric cardia, and gastric aspect of the gastroesophageal junction. However, those were far less dense and did not show active hemorrhage.

The patient required APC for the diffuse hemorrhagic PHG. However, our supply of argon was depleted at the time. We decided to coagulate the diseased mucosa with spray coagulation, which is, in principle, a similar technique to APC but does not require argon.

The patient was administered injection hyoscine to reduce motility. A cautery-enhanced snare with a 20-mm loop was used with an electrosurgical unit (Vio300D; Erbe, Germany) in spray coagulation mode (effect 2, 45 W) (Video 1, available online at www.igiejournal.org). The sheath of the snare was protruded a few millimeters outside the endoscope and kept in vision, and the snare itself was protruded 5 to 10 mm outside the sheath titrating to endoscopic vision of the effect on the mucosa. We found that our initial control over the burn was suboptimal, so we added a cap to the endoscope tip, which yielded much better control over the distance from the mucosa and thus a more controlled burn. After satisfactory hemostasis was achieved, the patient was kept on beta-blockers. No further drop in hemoglobin was observed. An EGD performed 2 days later showed significant residual PHG; however, there was no active hemorrhage. High-risk areas were again subjected to spray coagulation. The patient was discharged home the next day.

An EGD performed 1 week later showed marked reduction of PHG with no hemorrhagic spots. However, significant mucosal erythema was still present (Fig. 1). By this time, we had procured argon, and a session of APC was done. The patient required no further sessions of APC therafter, and over a year later, has not had any further episodes of bleeding or hepatic encephaloapthy.

## Discussion

Electrosurgical noncontact thermoablation involves placing a high-voltage electrode close to tissue. The intervening gaseous medium ionizes and then heats up because of the huge voltage difference, forming a hot plasma spark and allowing flow of current. The thermal coagulation is mainly caused by the endogenous heating caused by the current flow in the tissue and to some extent exogenously by the temperature of the plasma. Once coagulum is formed, the tissue becomes a poor conductor of electricity, and the plasma spark ceases to exist in this region. Thus, the effect is limited to superficial tissues only. Conversely, contact-thermoablative waveforms carry the risk of deeper penetration of heat, risking perforation and ulceration when used over large areas as required in diffuse mucosal pathologies.

**Figure 2:**
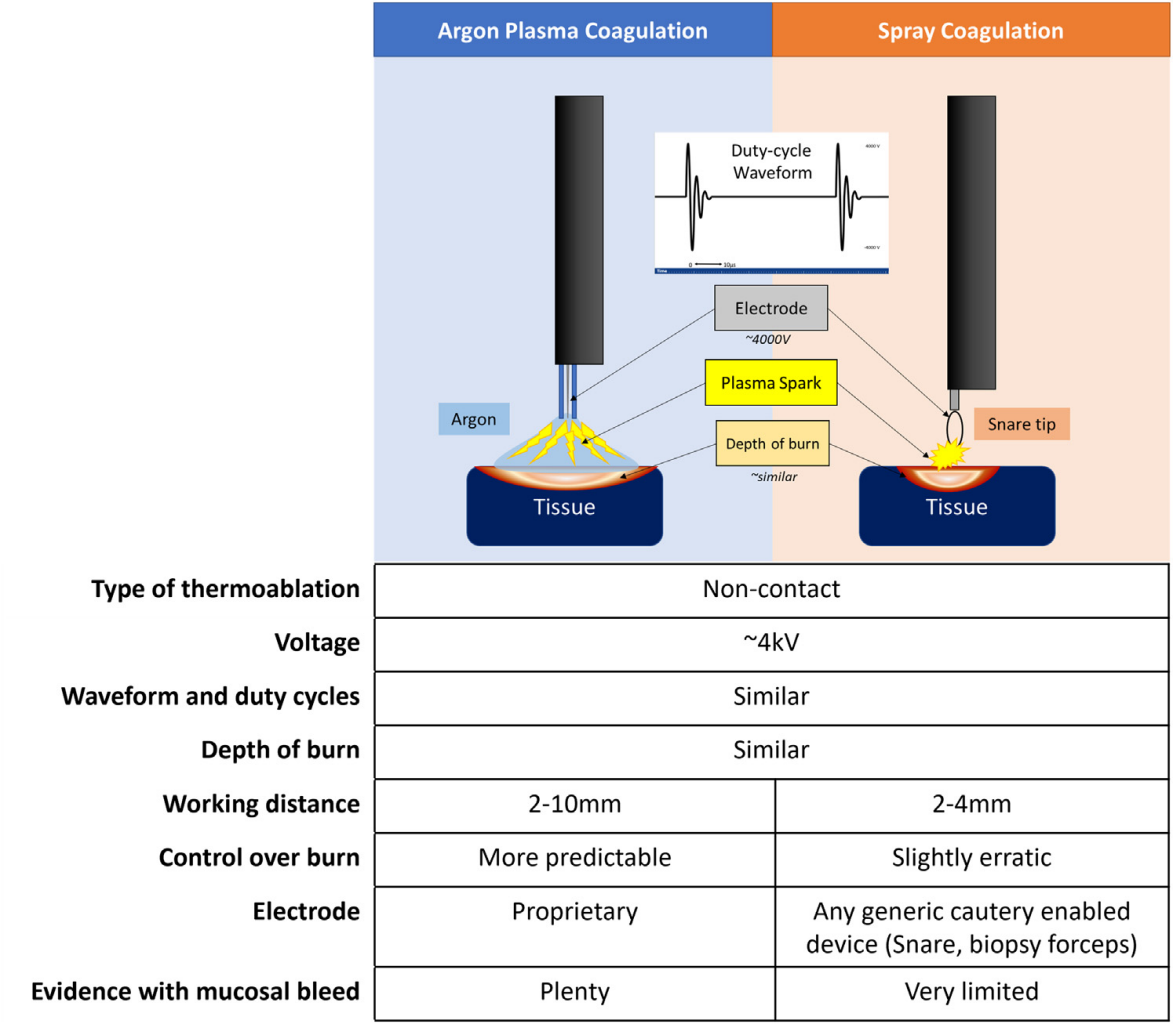
Schematic representation and comparison of technical aspects for both modalities.

Argon is an inert gas with a low breakdown voltage, which leads to formation of a diffuse, controlled plasma spark, and is thus the preferred mode for noncontact thermoablation in endoscopy. Spray coagulation works on similar principles of physics as APC.^3^ The high voltage (around 4000 V, same as APC) at the electrode tip relative to the mucosa leads to electrostatic breakdown of the intervening air (78% N_2_, 21% O_2_) instead of argon, forming a plasma spark. The electrosurgical unit crest factor waveforms for both are also similar, generated by bursts of high voltage that quickly dampens as current stops flowing. Also, on endoscopic view, both seem to achieve a similar effect on the mucosa (Fig. 2). The cautery-enhanced snare acted as the electrode in our case. Spray coagulation seems to achieve a similar effect as APC, albeit with a reduced working distance. In the absence of argon, spray coagulation thus became our next best choice for superficial ablation.

Spray coagulation has long been used to achieve hemostasis during surgeries. Recently, this technique was also used in third-space endoscopy for dissection of submucosal planes.^4^ Ex vivo evidence shows spray coagulation and APC can have similar efficacy of burn of the mucosal layer, reaching up to the submucosa but not damaging the muscularis propria.^5^ However, this has not yet been tested in human trials. Nevertheless, the technique has previously been used to achieve hemostasis during endoscopic necrosectomy and for angioectasias.^6^^,^^7^

We believed that control over the burn with spray coagulation was not as fine as that in APC; however, this may be a learning curve issue with the accessory. With the use of a cap, we were able to achieve better control over the tissue burn. Moreover, because the working distance is lower with spray coagulation, a cap helps prevent inadvertent contact with the mucosa.

The use of APC is quite limited in smaller endoscopy units, and the considerable premium for an APC-enabled electrosurgical unit over and above the base unit may not always be justified. Even in high-volume units, the recurring cost of single-use APC probes considerably increases the cost of the procedure. The use of spray coagulation may be a potential cost-cutting alternative. However, this needs to be compared in head-to-head trials. In conclusion, spray coagulation can be used as an alternative to APC in mucosal bleeding.

## Supplementary Material

Video 1 Endoscopic video of spray coagulation.
